# Research Waste in Randomised Control Trials of Spinal Manipulative Therapy for Chronic Low Back Pain: Evidence From Trial Sequential Analysis

**DOI:** 10.1002/ejp.70270

**Published:** 2026-04-06

**Authors:** Louise Elliott, Flora Chamberlain, Amin Yarahmadi, Sidney Rubinstein, Annemarie de Zoete, Martin Underwood

**Affiliations:** ^1^ Warwick Medical School University of Warwick Coventry UK; ^2^ University Hospitals Coventry and Warwickshire Coventry UK; ^3^ Department of Health Sciences, Faculty of Science Amsterdam Movement Sciences Research Institute, Vrije Universiteit Amsterdam the Netherlands; ^4^ Department of General Practice Erasmus Medical Center Rotterdam the Netherlands

## Abstract

**Background:**

Chronic low back pain is the leading cause of years lived with disability worldwide. Although spinal manipulation therapy (SMT) is recommended in clinical guidelines and supported by randomised clinical trials (RCTs), systematic review conclusions have not changed despite a growing evidence base. This trial sequential analysis evaluates the extent of research waste in this field by identifying when sufficient evidence had been gathered.

**Methods:**

We report a Trial Sequential Analysis using data from the Cochrane review on SMT. This methodological approach assesses whether statistically significant conclusions can be drawn from accumulating evidence by plotting Z‐curve graphs with monitoring boundaries; where the Z‐scores' fall indicates the benefit, harm or futility of the intervention. Trial Sequential Analysis estimates a required information size, analogous to optimum sample size in a single trial, based on the data. Our primary analysis explored whether conclusions have been drawn for SMT versus other conservative treatments for pain and functional status at 1 month.

**Results:**

We included, 60 RCTs (*N* = 10,312). For the effect of spinal manipulative therapy versus conservative treatments for pain at 1 month, the futility and information size boundary was crossed in 2004, and in 2002 for functional status. At longer follow‐up times, these boundaries were reached at similar dates.

**Conclusions:**

RCTs from as early as 2002 have not added to our understanding of the effectiveness of spinal manipulative therapy for chronic low back pain and functional status and therefore constitutes research waste. Conducting further RCTs in this field without a novel research question should be avoided.

**Significance Statement:**

This important research quantifies research waste in the field of chronic low back pain, a condition affecting billions worldwide. We advocate for trials addressing novel questions to ensure finite resources go towards effective treatments rather than producing redundant evidence; upholding research ethics. Highlighting innovative use of trial sequential analysis will provide a model for visually and quantitatively demonstrating research waste which if utilised across disciplines can ensure research prioritisation, increased production of high‐value evidence‐based medicine for patients, ultimately improving outcomes.

## 1 Introduction

Many interventions exist for the treatment and management of low back pain, including spinal manipulative therapy (SMT), which is a form of hands‐on mobilisation and/or manipulation of the spine, designed to treat symptoms of low back pain. This involves both high and low velocity movements of the spine, which is thought to work on the biomechanics and neurophysiology of the spinal lesion to improve pain and functional status (Bialosky et al. 2009; Coronado et al. 2012; Wong et al. 2015; Xia et al. 2017). It is delivered by a variety of healthcare professionals including chiropractors, osteopaths or physiotherapists. It is a recommended treatment as a stand‐alone treatment or as part of a broader package approach in many guidelines for chronic low back pain (National Institute for Health and Care Excellence (NICE) 2020; World Health Organisation 2023; Zaina et al. 2023; Zhou et al. 2024).

Globally, low back pain is the leading cause of years lived with disability (World Health Organisation 2022). By 2050, over 800 million people are likely to have back pain; signifying a need for research into interventions to treat this debilitating condition (Global Burden of Disease Collaborators 2023). Evidence is needed to inform choice for patients, clinicians, healthcare organisations and other stakeholders for the treatment and management of low back pain.

Several high‐quality meta‐analyses have examined the effectiveness of SMT for chronic low back pain. A 2011 Cochrane review (26 randomised clinical trials [RCTs], *N* = 6070) concluded that there was high‐quality evidence suggesting that SMT provides statistically significant but not clinically relevant improvements in pain and functional status compared to other conservative treatments (Rubinstein et al. 2011). An update to that review, published in 2019 (47 RCTs, 9211 participants) came to similar conclusions, as did the 2026 Cochrane review (76 RCTs, *N* = 11,866) (de Zoete et al. 2026; Rubinstein et al. 2019). Conclusions about SMTs effectiveness for pain relief and improvement of functional status for the treatment and management of chronic low back pain are largely unchanged over time.

This begs the question whether our time and resources for researching the treatment and management of chronic low back pain couldn't be better used. Research waste is unjustifiable from a clinical, ethical and financial perspective because little benefit can be drawn from the outcomes. It appears that there is a substantial amount of research waste in the field of SMT for chronic low back pain; the extent of this waste prompted a call‐to‐action (Aspinall et al. 2024). Two key strategies to avoid research waste include: (1) preventing unnecessary duplication of research and (2) ensuring that the needs of policymakers and practitioners are addressed (Wolfenden et al. 2015). Although numerous RCTs have investigated SMT for chronic low back pain over recent decades, it would appear based upon the conclusions of these Cochrane reviews published in recent decennia have added little to our understanding. Therefore, we aimed to determine the extent of research waste in the field of SMT for chronic low back pain by determining the point in time trials became redundant using trial sequential analysis (TSA). Our objective was to determine when RCTs on SMT for chronic low back pain did not add new information about effectiveness.

## 2 Methods

### 2.1 Study Design: Meta‐Epidemiological Study

For our primary analysis, we included data for SMT versus other conservative treatments assessing both pain reduction and improvement of functional status. The primary end point was one‐month. Secondary end points included follow‐ups at three‐, six‐ and twelve‐months. We carried out a secondary analysis using SMT versus sham SMT/placebo treatments comparisons at similar end points.

We prospectively registered this study protocol publicly at Open Science Framework; with registration DOI https://doi.org/10.17605/osf.io/cxa7q.

We used data from 60 RCTs included in the forthcoming Cochrane review *SMT for Chronic Lower Back Pain* for comparisons of SMT against other conservative treatments and sham SMT/placebo treatments at one‐ three‐ six‐ and twelve‐month follow‐up times for pain and functional status outcomes, as per the original review analysis follow‐up time‐points. No new literature search or risk of bias assessments were performed for our analyses. The data sets that we have used can be accessed through the Cochrane meta‐analysis on SMT for chronic LBP (de Zoete et al. 2026).

### 2.2 Trial Sequential Analysis

Trial sequential analyses were performed to assess the cumulative strength of existing evidence to determine whether sufficient data exist to draw conclusions regarding the benefit, harm or futility of SMT with other conservative treatments and sham SMT/placebo treatments.

**FIGURE 1:**
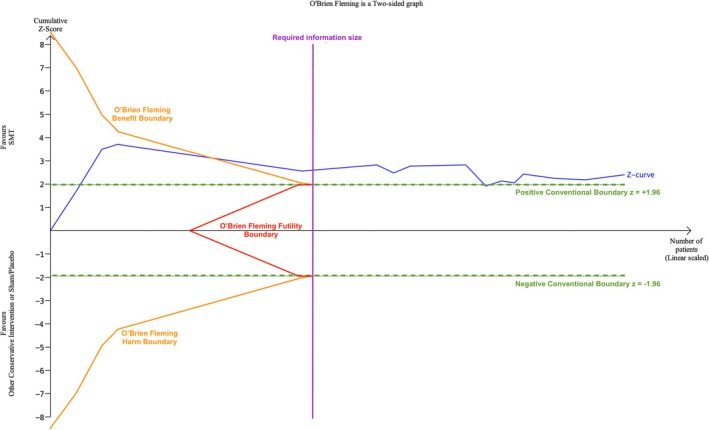
Schematic of Trial Sequential Analysis. The blue line represents the cumulative Z‐score, in which the Cochrane RCTs add sequentially in chronological order. The orange curves indicate the O'Brien–Fleming trial sequential monitoring boundaries for benefit (positive) and harm (negative), while the red line denotes the futility boundary (where the Z‐curve would have to land within to result in a conclusion of a futile intervention). The purple vertical line represents the required information size. The green dashed lines indicate the conventional boundaries for statistical significance corresponding to a two‐sided *p*‐value of 0.05 (the Z‐score would have to either land either > + 1.95 or < −1.95 to demonstrate conventional statistical significance). The schematic illustrates a scenario in which the cumulative Z‐curve crosses the boundary for benefit before the required information size is reached and remains above the conventional significance boundary past the required information size.

TSA uses cumulative meta‐analysis software to quantify statistically significant thresholds for the benefit, harm or futility of an intervention prior to the required information size being reached; determined through advanced probability theorems which allow control of type I and II error rates using Lan‐DeMets methodology and demonstrated by O'Brien‐Fleming monitoring boundaries. Illustrated in Figure 1, trials were added sequentially to the meta‐analysis and expressed as cumulative z‐scores, generating a chronologically ordered Z‐curve. Crossing the O'Brien‐Fleming boundaries for benefit or harm, or crossing into futility prior to required information size being reached (or crossing the conventional significance boundaries once the required information size was exceeded), was interpreted as evidence that further trials are unlikely to alter the estimated effect of SMT compared to other conservative treatments and sham SMT/placebo treatments (Figure 1) (Brok et al. 2008; De Cassai et al. 2021; Kang 2021; Pogue and Yusuf 1998, 1997; Thorlund et al. 2009, 2017; Wetterslev et al. 2008, 2009). It is important to note that clinically important differences cannot be discerned from these diagrams and were used solely to demonstrate when we had sufficient data to determine a statistically significant conclusion about the effectiveness of the interventions.

Required information size in context of TSA is akin to sample size calculations that are required to ensure a single RCT is adequately powered; it determines a required sample size for meta‐analysis from all included RCTs that will provide reliable statistical inference. This software allows calculation of the required information size using a random‐effects model and therefore adjusts the value according to the variation of the included data.

Using the data from RCTs included in the Cochrane systematic review published in early 2026, we did random effects (Der Simonian‐Laird) trial sequential meta‐analyses. The TSA tool developed by the Copenhagen Clinical Trials Unit was used for these analyses and we defined our Type I and Type II error rates as *α* = 0.05 and *β* = 0.1 respectively (Thorlund et al. 2017).

We extracted the author, publication date, mean, standard deviation and number of participants from the 2025 Cochrane review data for each outcome and time‐point into the TSA software; a cumulative Z‐curve was plotted by trial publication date. Trial Sequential analysis could not be created for SMT versus sham SMT/placebo treatments at three‐month follow‐up for functional status due to the required information size being crossed by the first trial. Pain and functional status outcome analyses at 12‐month follow‐up for SMT versus sham SMT/placebo treatments could not be carried out as there is only one trial in this comparison.

We converted functional status scores to a 100‐point scale by reviewing each of the included RCTs papers to identify the specific functional measure scales used. For measures with a maximum score of less than 100, we mathematically converted them. The standardisation of these scores was necessary to ensure compatibility with the TSA software; previous evidence from back pain research has justified this conversion step to improve data use. (Thorlund et al. 2017; Wewege et al. 2022).

## 3 Results

**FIGURE 2:**
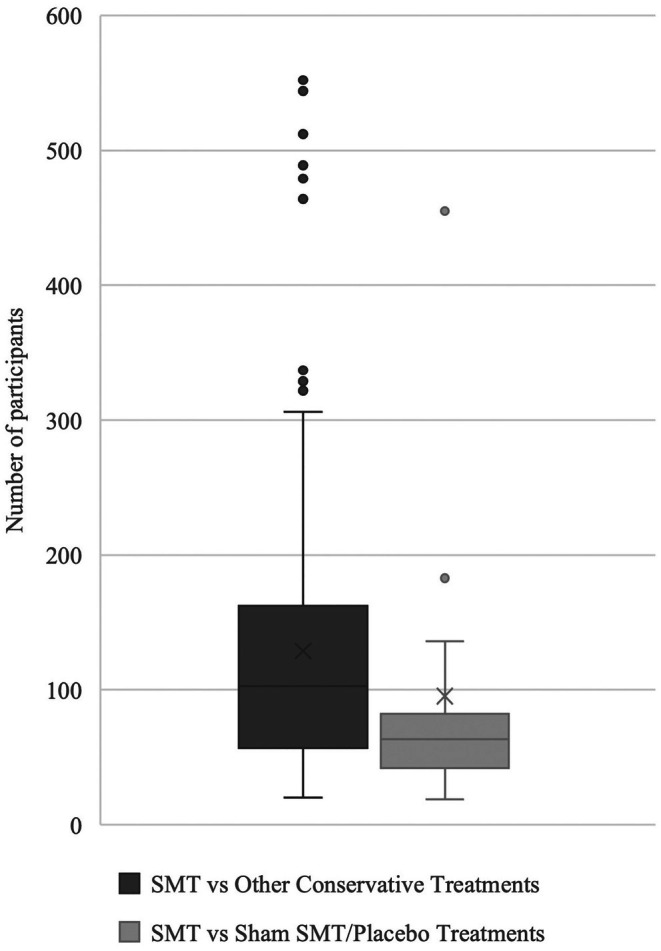
Box and whisker plot of number of participants from pain and function outcomes at all time frames for both primary and secondary analyses: SMT versus Other Conservative Treatments and SMT versus Sham SMT/Placebo Treatments.

In total, 60 RCTs (*N* = 10,312) carried out between 1989 and 2024 were included in our analyses. The median participant size for comparisons of SMT versus other conservative treatments was 103 with QRR = 57–162. The median participant size for comparisons of SMT versus sham SMT/placebo treatments was 63.5 with QRR = 44–78. We generated box and whisker graphs for both of these comparisons (Figure 2).

### 3.1 Primary Analyses

#### 3.1.1 SMT Versus Other Conservative Treatments

##### 3.1.1.1 Pain

**FIGURE 3:**
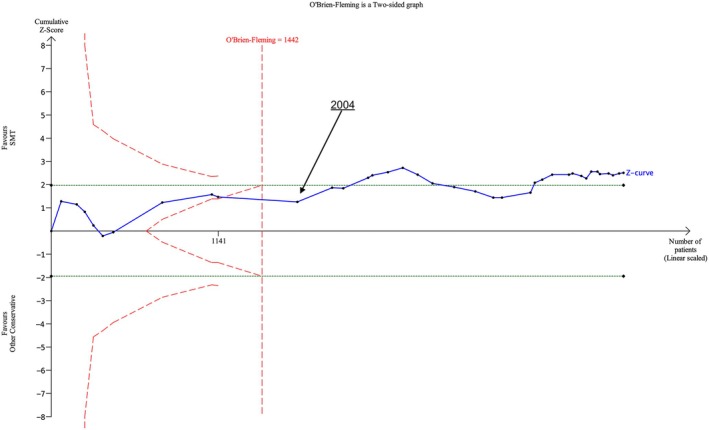
Trial sequential analysis of pain at 1 month follow‐up by date for SMT versus other conservative treatments.

Data from 37 RCTs (*N* = 3914) were analysed for one‐month follow‐up. The futility boundary was crossed and the required information size exceeded in 2004 with the inclusion of the UK BEAM trial (UK BEAM Trial Team 2004). An additional 27 RCTs (*N* = 2229) were identified following this publication, with the most recent publication in 2024 (Figure 3). At subsequent follow‐up measurements, the futility boundary and the required information size were crossed in 2004 for three‐month follow‐up and in 2002 at six‐ and 12‐month follow‐up (Figure S1).

##### 3.1.1.2 Functional Status

**FIGURE 4:**
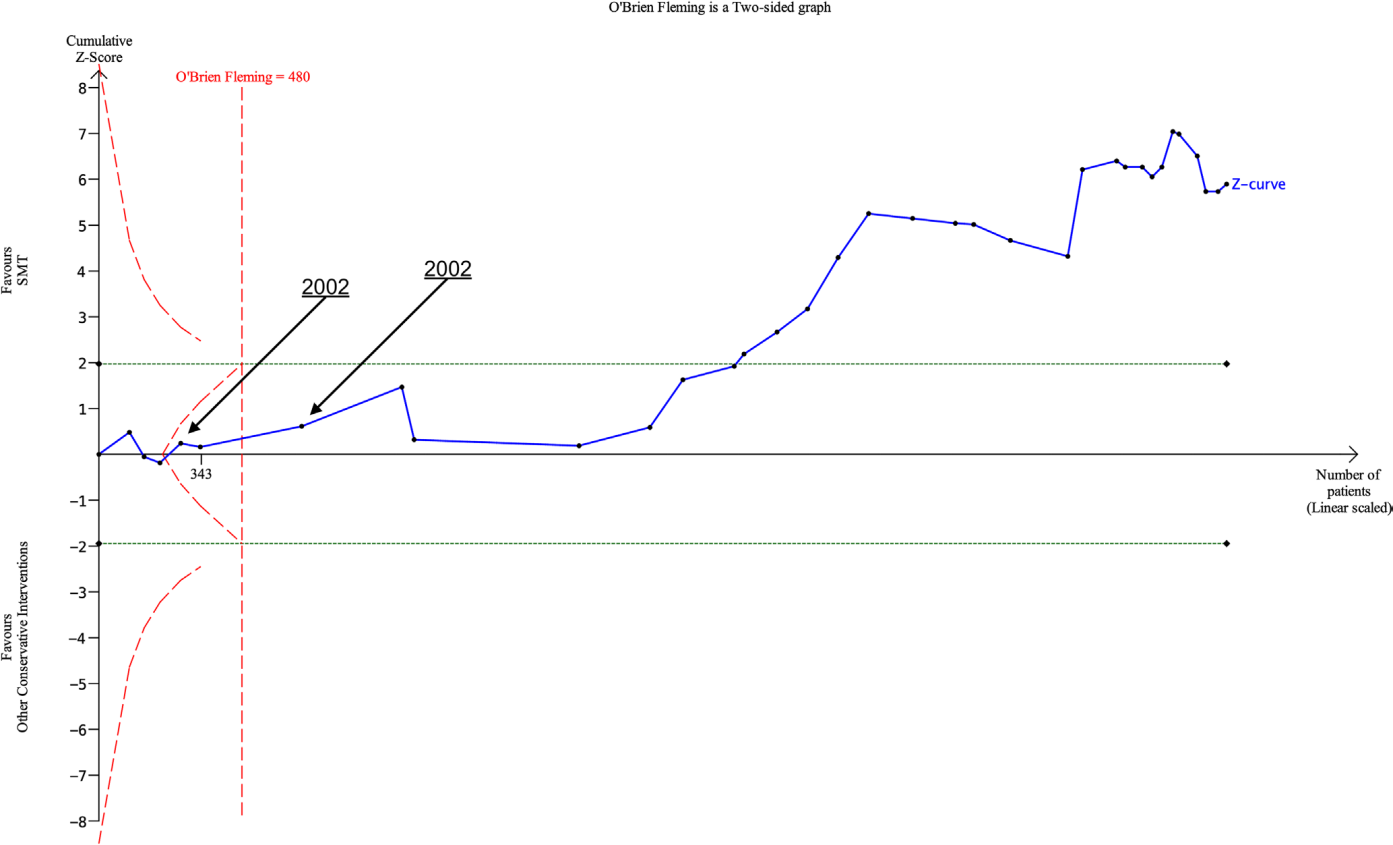
Trial sequential analysis of function at 1 month follow‐up by date for SMT versus other conservative treatments.

Data from 34 RCTs (*N* = 3778) were analysed for one‐month follow‐up. The fourth included RCT published in 2002 took us past the futility boundary with inclusion of the Hsieh et al. trial; a further two trials carried out in this same year took us past the required information size (Hsieh et al. 2002). A total of 28 additional RCTs with a cumulative 3097 participants were included in RCTs after 2002. (Figure 4). Both futility and the required information size boundaries were exceeded with functional status outcomes at the later follow‐up times of three‐ and six‐months in 2004 and 2002 respectively. At 12‐months follow‐up, the required information size was exceeded in 2002 (Figure S2).

### 3.2 Secondary Analyses

#### 3.2.1 SMT Vs. Sham SMT/Placebo Treatments

##### 3.2.1.1 Pain

**FIGURE 5:**
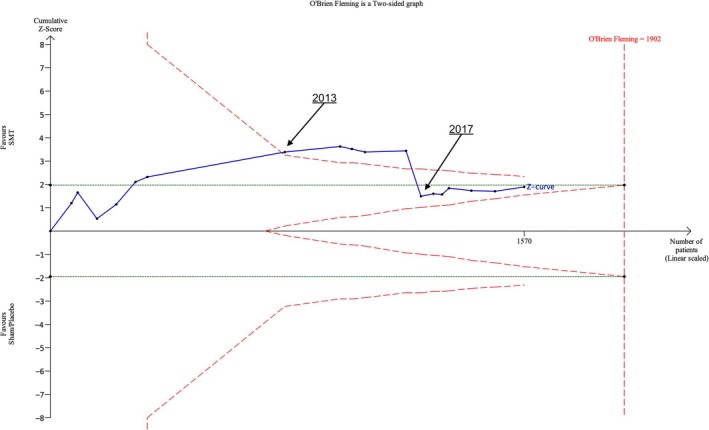
Trial sequential analysis of pain outcome at 1 month follow‐up by date for SMT versus Sham SMT/placebo treatments.

A total of 16 RCTs (*N* = 1570) were analysed. At one‐month follow‐up, the O'Brien‐Fleming benefit boundary was crossed in 2013 by inclusion of Licciardone et al.; a further four RCTs remain above the benefit boundary until 2017, when the Z‐curve falls back below the benefit boundary and remains outside O'Brien‐Fleming futility boundary (where the Z‐curve would lie between the v‐shaped lines) and conventional boundaries (where the Z‐score would have to lie above the green dashed lines) for the remainder of the analysis (Figure 5) (Licciardone et al. 2013). At three‐month follow‐up, futility boundaries are crossed and required information size is exceeded by 2013. The futility boundary was crossed in 2011. At 12‐month follow‐up, the required information size was exceeded by the first trial, and therefore O'Brien‐Fleming boundaries could not be created by the TSA software. (Figure S3).

##### 3.2.1.2 Functional Status

**FIGURE 6:**
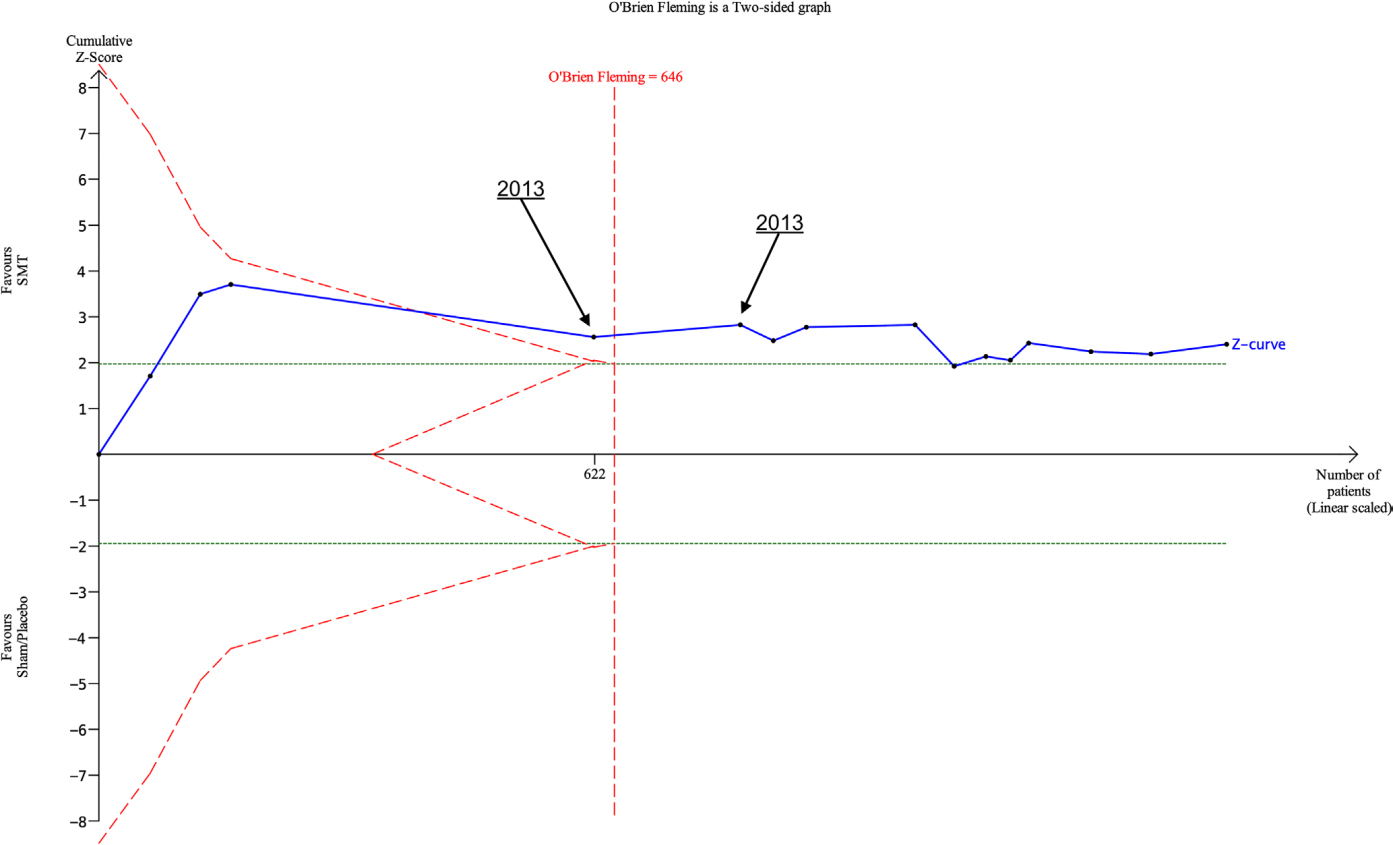
Trial sequential analysis of function outcomes at 1 month follow‐up by date for SMT versus Sham SMT/placebo treatments.

At one‐month follow‐up time we cross the O'Brien‐Fleming boundary for benefit of SMT with inclusion of Licciardone et al. published in 2013, with one further trial in 2013 taking us past the required information size (Licciardone et al. 2013). A further eight RCTs up until 2024 were carried out past this point; these 10 further RCTs involved 611 participants (Figure 6). Follow‐up at 3 months and 6 months demonstrates similar patterns (Figure S4). A graph could not be created by the software for 12‐month follow‐up as only one trial investigated outcomes at this time point.

### 3.3 Post Hoc Analyses

**FIGURE 7:**
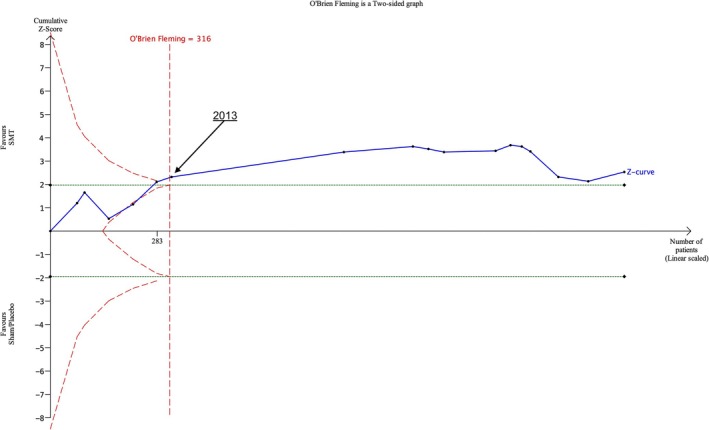
Post hoc Trial sequential analysis of pain outcomes at 1 month follow‐up by date for SMT versus Sham SMT/placebo treatments with 2017 trial removed from the analysis.

In the comparison of SMT with sham SMT/placebo at the one‐month follow‐up for pain outcomes, our planned analyses did not follow the expected pattern of a monotonic increase in statistical significance. The Z‐curve dropped below the O'Brien‐Fleming boundary in 2017 after it had been crossed by five data points. We did further post hoc analyses exploring the effect of removing one trial with an unusually large and potentially untrustworthy effect size. The mean difference of the trial in 2017 that resulted in the drop of the Z‐curve was found to be −46.80 (−52.42, −41.18), with the average of all other included RCTs to be −3.51 (−6.28, −0.74). Once we identified this, we removed the RCT from our TSA for this comparison in the first instance and resulted in a dramatically altered graph, the benefit boundary crossed and required information size exceeded (which had also reduced from 1902 participants to 316) in 2012. (Figure 7). O'Brien‐Fleming boundaries were crossed and required information size (which also reduced) was exceeded at earlier time‐points in other analyses with this trial excluded.

## 4 Discussion

Sufficient data had been accumulated to provide a statistically significant conclusion about the utility of SMT decennia ago. Our trial sequential analyses demonstrate that O'Brien‐Fleming boundaries were crossed in early 2002 for both pain and functional status outcomes across all follow‐up times, as well as the required information size being exceeded in almost all analyses at approximately the same time. Importantly, this demonstrates substantial research waste in the area of SMT for these outcomes for the treatment and management of chronic low back pain. A substantial number of RCTs have been carried out beyond the point at which a definitive conclusion about the benefit of SMT had already been reached. The most striking example is the TSA for SMT versus other conservative treatments for functional status outcomes at one‐month follow‐up by date, where 28 additional RCTs were conducted after reaching statistical significance in 2002, with subsequent RCTs up until 2024, accruing a further 3097 participants. These findings emphasise the large extent of unnecessary research conducted in this area, despite the availability of conclusive evidence regarding SMTs effectiveness. This provides a striking and quantifiable message to SMT researchers: any further investigation into the effectiveness of SMT for chronic low back pain is wasteful.

An important limitation is the lack of accounting for the risk of bias. Specifically, we did not examine whether risk of bias domains would have impacted the time frame we identified. That said, it is highly questionable to whether this is relevant. Meta‐epidemiological studies which have examined the association between effect size and RoB for exercise and SMT suggest no appreciable association (Innocenti et al. 2023; Nim et al. 2026). Furthermore, those findings are consistent with the 2026 Cochrane SMT review, which found no impact of the RoB in extensive sensitivity analyses (de Zoete et al. 2026). In short, this would seem to reinforce the validity of our results.

Our post hoc analyses highlighted that inclusion of just one RCT with outlying results can affect the conclusions of a meta‐analysis. The removal of this one trial in our post hoc analyses resulted in a substantial reduction in the required information size. Typically, removing one trial should either cause little change or increase the required information size and shift the crossing of the O'Brien‐Fleming boundaries to require further RCTs to come to a conclusion, not fewer (Wetterslev et al. 2009). Evidence from related fields has found that untrustworthy trials with outlying results can affect systematic review conclusions (Chamberlain et al. 2025; O'Connell et al. 2023).

Our study is consistent with a recent commentary highlighting the pervasive research waste in SMT studies, arising from some of the issues which we addressed, such as small sample sizes, methodological flaws, poor reporting and heterogeneous systematic reviews. That study stresses that both clinical and mechanistic SMT research often lack rigour, with inadequate blinding, limited external validity and insufficient exploration of clinically relevant mechanisms. The paper issued a call‐to‐action for SMT researchers to improve study design, conduct and reporting, offering key recommendations and resources to enhance future research quality (Aspinall et al. 2024).

### 4.1 Strengths and Limitations

TSA is a statistically complex tool that comes with some drawbacks and criticisms. The interpretation of TSA should proceed with caution as crossing of boundaries may happen by chance due to the *p*‐value; however, it is argued that TSA provides cautious results (Jennison and Turnbull 1999; Kang 2021; Reboussin et al. 2000). Importantly, all outcomes, particularly with clinical application, must be viewed in the context of their application and whether findings are relevant to, and safe for, patients. The main clinical results of the Cochrane review from which these data have been drawn detail the actual comparative effectiveness for studies comparing SMT to other conservative treatments or to Sham/placebo controls (de Zoete et al. 2026).

The Cochrane Scientific Committee advises against the use of TSA in primary meta‐analyses due to several limitations. These include its inability to analyse multiple outcomes with different thresholds, its reliance on binary outcomes, as well as the risk of reporting bias. Cochrane particularly discourages its use in meta‐analyses involving smaller RCTs or when assessing outcomes in practical clinical contexts (Thomas et al. 2024). We acknowledge Cochrane's reservations; however, their concerns do not pertain to our use of TSA. It is not employed as a primary meta‐analytic tool but as a supplementary method to assess the robustness of existing findings. TSA is applied for secondary analysis of previously conducted and methodologically robust meta‐analyses. We have ensured that our preregistered protocol specified parameters for our TSA analyses, to reduce risk of reporting bias (Shamseer et al. 2015). Their concerns raised are therefore not applicable to our analytical framework and do not compromise the relevance or validity of our findings.

Whilst our findings indicate that we have reached definitive conclusions regarding whether SMT is superior to either other conservative treatments or sham SMT/placebo treatments, this does not infer equivalence between treatments. Notably, none of the included studies were designed as equivalence RCTs.

Any new RCTs of spinal manipulative therapy need to have a clearly identified novel research question and avoid conducting additional RCTs in a similar way to those previously performed, especially small RCTs with a high risk of bias. The findings of this study only related to low back pain. There is still a need, as identified in the NICE chronic pain guideline for new trials of manual therapy for some other painful musculoskeletal disorders (National Institute for Health and Care Excellence [NICE] 2020). Future SMT trials need to clarify how SMT works—whether through spinal biomechanics, neuromuscular responses or central pain modulation pathways—and identify which clinical subgroups respond best. To achieve this, studies should align the proposed mechanism with their inclusion criteria and outcome measures; for example, if restricted range of motion is a key target, participants should present with this limitation and range of motion improvement should be included as a primary outcome.

### 4.2 Future Directions

Our research provides evidence of the large extent of research waste in this area, something that has been a concern for many years. Specific guidance on how to improve the relevance of new research is available in other academic literature (Aspinall et al. 2024). Thousands of participants have been involved in RCTs after definite conclusions have been drawn about the effectiveness of SMT for pain and functional status outcomes in chronic low back pain. Future RCTs in this area that do not address new research questions would be financially and morally problematic.

This problem of research waste in low back pain research is not limited to trials of SMT. In 2012 Ferriera found that further trials of exercise for low back pain were unlikely to be informative (Ferreira et al. 2012). Ravestyn in 2020 found that in spite of this recommendation the proportion of trials for chronic low back that we of exercise treatments had continued to increase (van Ravesteyn et al. 2021). Our own work from 2025, using the trial sequential approach found that for trials of exercise for chronic low back pain superiority boundaries for both pain and disability were passed in 2014 (Chamberlain et al. 2025). Similarly, this paper continues to provide evidence and recommendations, mirroring the overarching goals of these works; reducing research waste by ensuring methodological rigour in further research and investigate knowledge gaps. How to enforce this, however, is very problematic as and risks prematurely discouraging research in areas of uncertainty and heterogeneity.

A major challenge in biomedical research is the frequent duplication of studies without meaningful contributions to clinical practice (Cooper et al. 2005). This issue is often driven by the pressures of career progression, where individuals—particularly those in clinical careers—may be incentivised to publish for career advancement rather than to address critical gaps in knowledge or improve patient outcomes (Altman 1994). It is imperative that future research efforts prioritise a thorough understanding of existing evidence to identify areas where further research would be most valuable, rather than conducting redundant RCTs.

## 5 Conclusions

Randomised controlled trials from as early as 2002 and onward have not added to our understanding of the effectiveness of SMT for chronic low back pain and functional status and therefore constitute research waste. Conducting additional RCTs in a similar way to those previously performed should be avoided. Future RCTs of SMT need to have a clearly identified novel research question if they are to be viewed as relevant.

## Author Contributions

L.E. and F.C. are joint first authors. M.U. initiated the study. F.C., L.E., A.Y. and M.U. designed the study. F.C. and L.E. conducted data analysis with statistical support from A.Y. provided. All authors were involved in data interpretation and involved in writing of the manuscript, led by L.E. M.U. is the guarantor and confirms the manuscript is an accurate and honest account of the study being reported and that no important aspects of the study have been omitted, with any discrepancies explained.

## Conflicts of Interest

Martin Underwood is chief investigator or co‐investigator on multiple previous and current research grants from the UK National Institute for Health Research and is a co‐investigator on grants funded by the Australian NHMRC and Norwegian Research Council. He is a director and shareholder of Clinvivo Ltd that provides electronic data collection for health services research. He receives some salary support from University Hospitals Coventry and Warwickshire. He is a co‐investigator on two current and one completed NIHR funded studies that have, or have had, additional support from Stryker Ltd. He has accepted travel expenses for speaking at academic meetings. Annemarie de Zoete works in private practice as a Chiropractor. All other authors declare no conflicts of interest.

## Supporting information


**Figure S1:** Trial sequential analysis of pain outcomes at 3‐, 6‐ and 12‐months follow‐up by date for SMT versus Other Conservative Treatments.
**Figure S2:** Trial sequential analysis of functional status outcomes at 3‐, 6‐ and 12‐months follow‐up by date for SMT versus Other Conservative Treatments.
**Figure S3:** Trial sequential analysis of pain outcomes at 3 and 6‐months follow‐up by date for Sham SMT/Placebo treatments. Trial sequential analysis could not be performed on data at 12‐months as there was only 1 included trial.
**Figure S4:** Trial sequential analysis of functional status outcomes at 3 and 6‐months follow‐up by date for Sham SMT/Placebo treatments. Trial sequential analysis could not be performed on data at 12‐months as there was only 1 included trial.
