# Supplementary material for: Research Waste in Randomised Control Trials of Spinal Manipulative Therapy for Chronic Low Back Pain: Evidence From Trial Sequential Analysis
Source: Eur J Pain. 2026 Apr 6;30(4):e70270. doi: 10.1002/ejp.70270 (PMC13053911; doi:10.1002/ejp.70270)
Supplement: Supplementary file 1 — Figure S1: Trial sequential analysis of pain outcomes at 3‐, 6‐ and 12‐months follow‐up by date for SMT versus Other Conservative Treatments. Figure S2: Trial sequential analysis of functional status outcomes at 3‐, 6‐ and 12‐months follow‐up by date for SMT versus Other Conservative Treatments. Figure S3: Trial sequential analysis of pain outcomes at 3 and 6‐months follow‐up by date for Sham SMT/Placebo treatments. Trial sequential analysis could not be performed on data at 12‐months as there was only 1 included trial. Figure S4: Trial sequential analysis of functional status outcomes at 3 and 6‐months follow‐up by date for Sham SMT/Placebo treatments. Trial sequential analysis could not be performed on data at 12‐months as there was only 1 included trial. [file EJP-30-0-s001.pptx]

## Slide 1
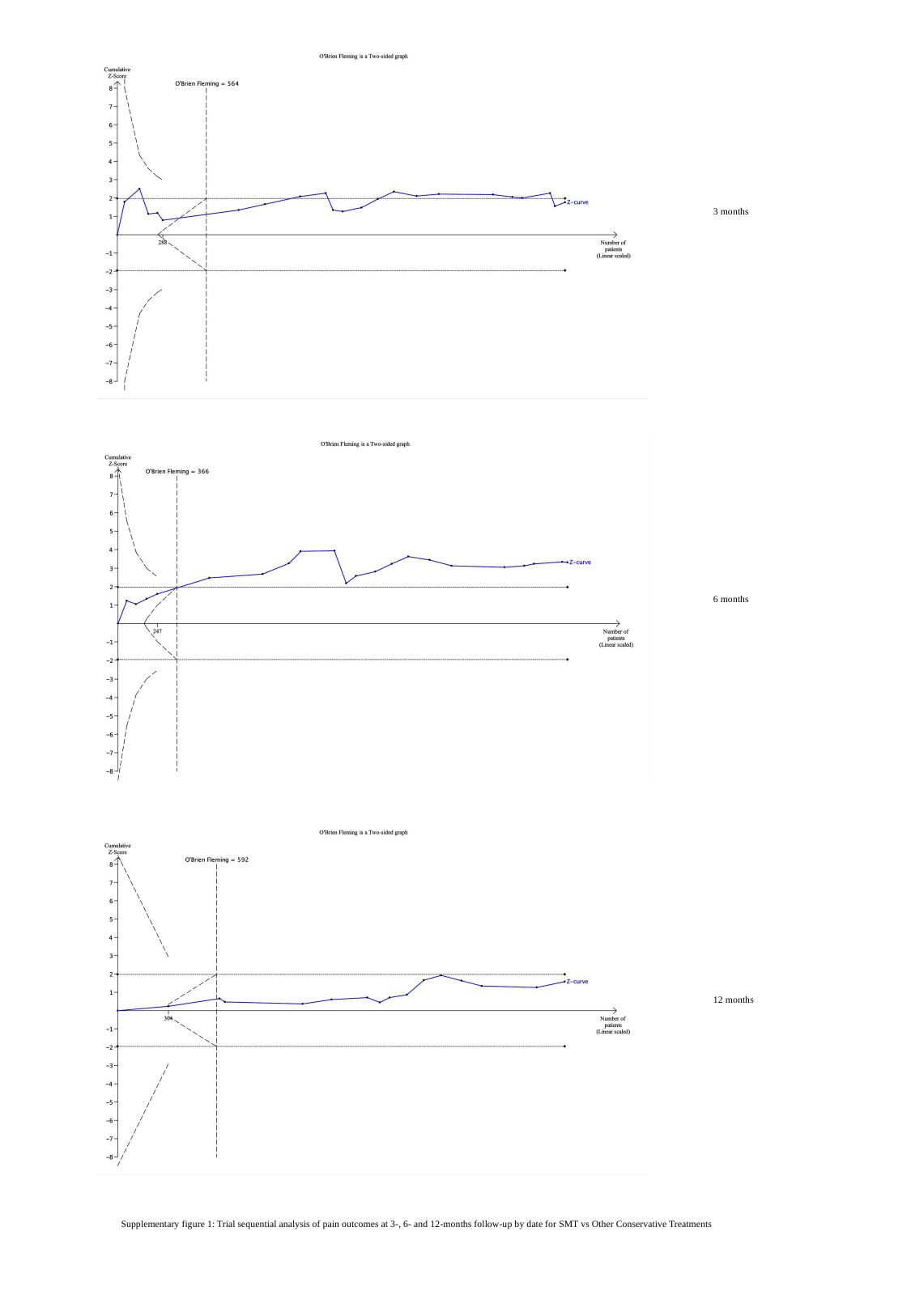

3 months
6 months
12 months
Supplementary figure 1: Trial sequential analysis of pain outcomes at 3-, 6- and 12-months follow-up by date for SMT vs Other Conservative Treatments

## Slide 2
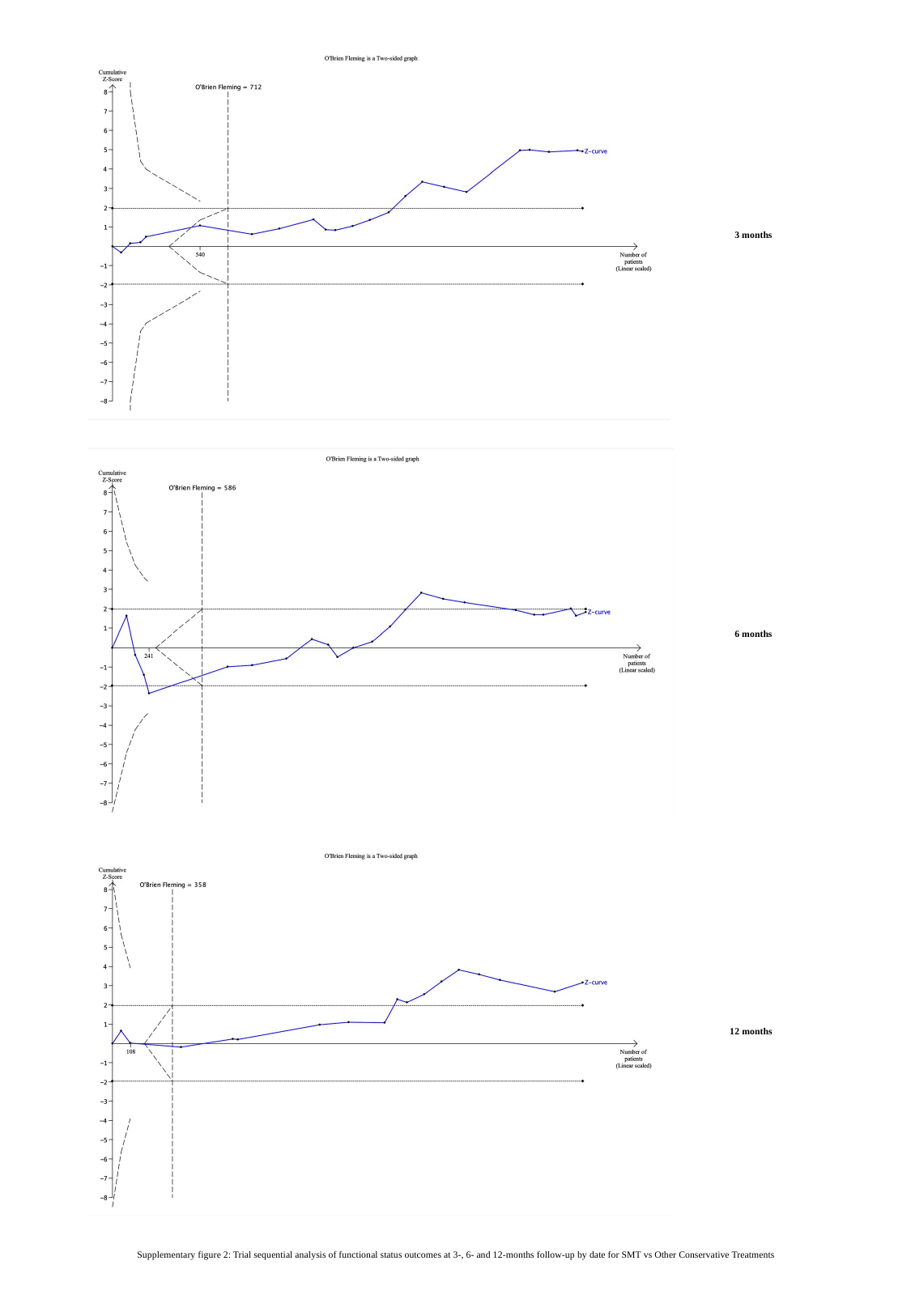

3 months
6 months
12 months
Supplementary figure 2: Trial sequential analysis of functional status outcomes at 3-, 6- and 12-months follow-up by date for SMT vs Other Conservative Treatments

## Slide 3
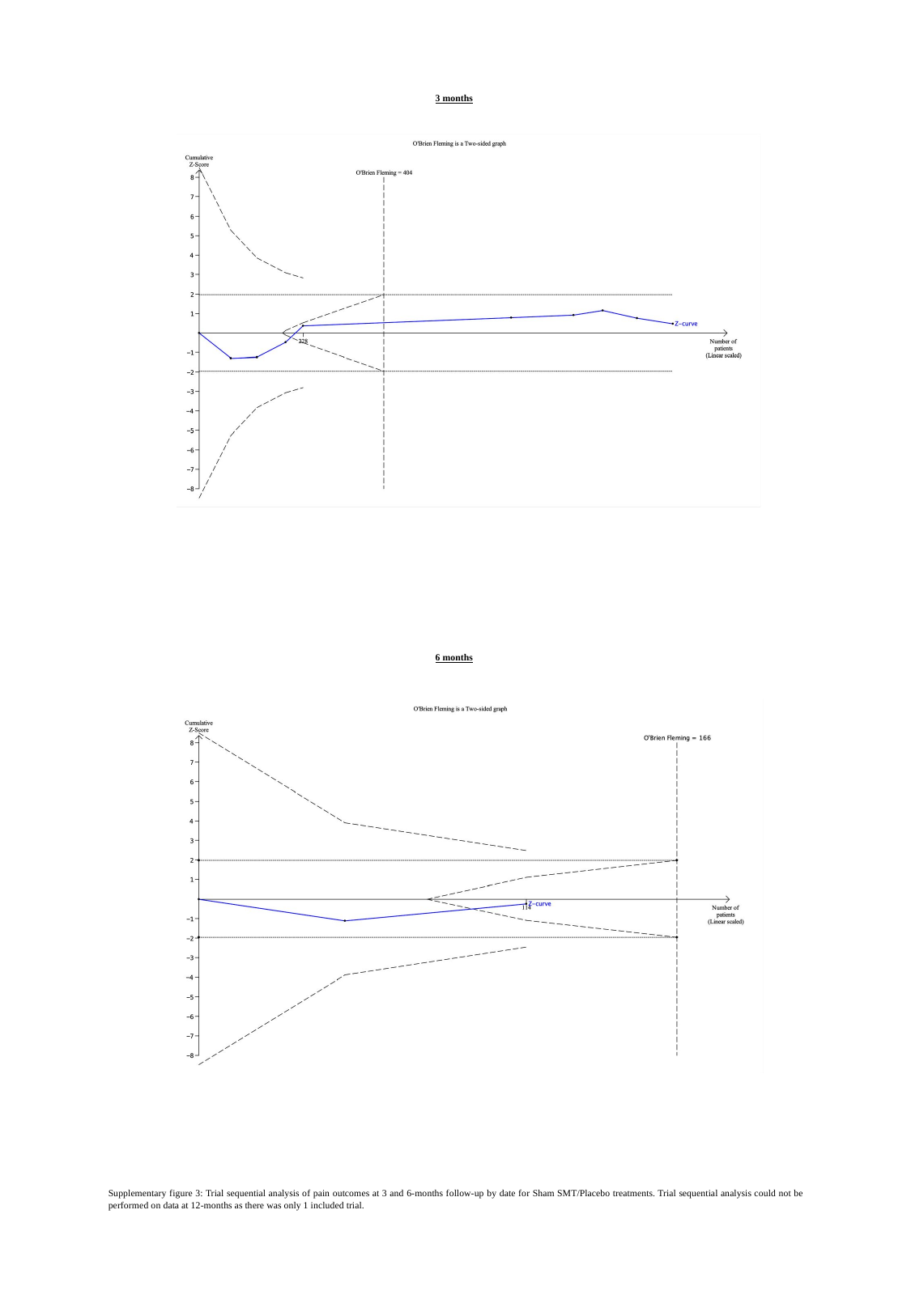

3 months
6 months
Supplementary figure 3: Trial sequential analysis of pain outcomes at 3 and 6-months follow-up by date for Sham SMT/Placebo treatments. Trial sequential analysis could not be performed on data at 12-months as there was only 1 included trial.

## Slide 4
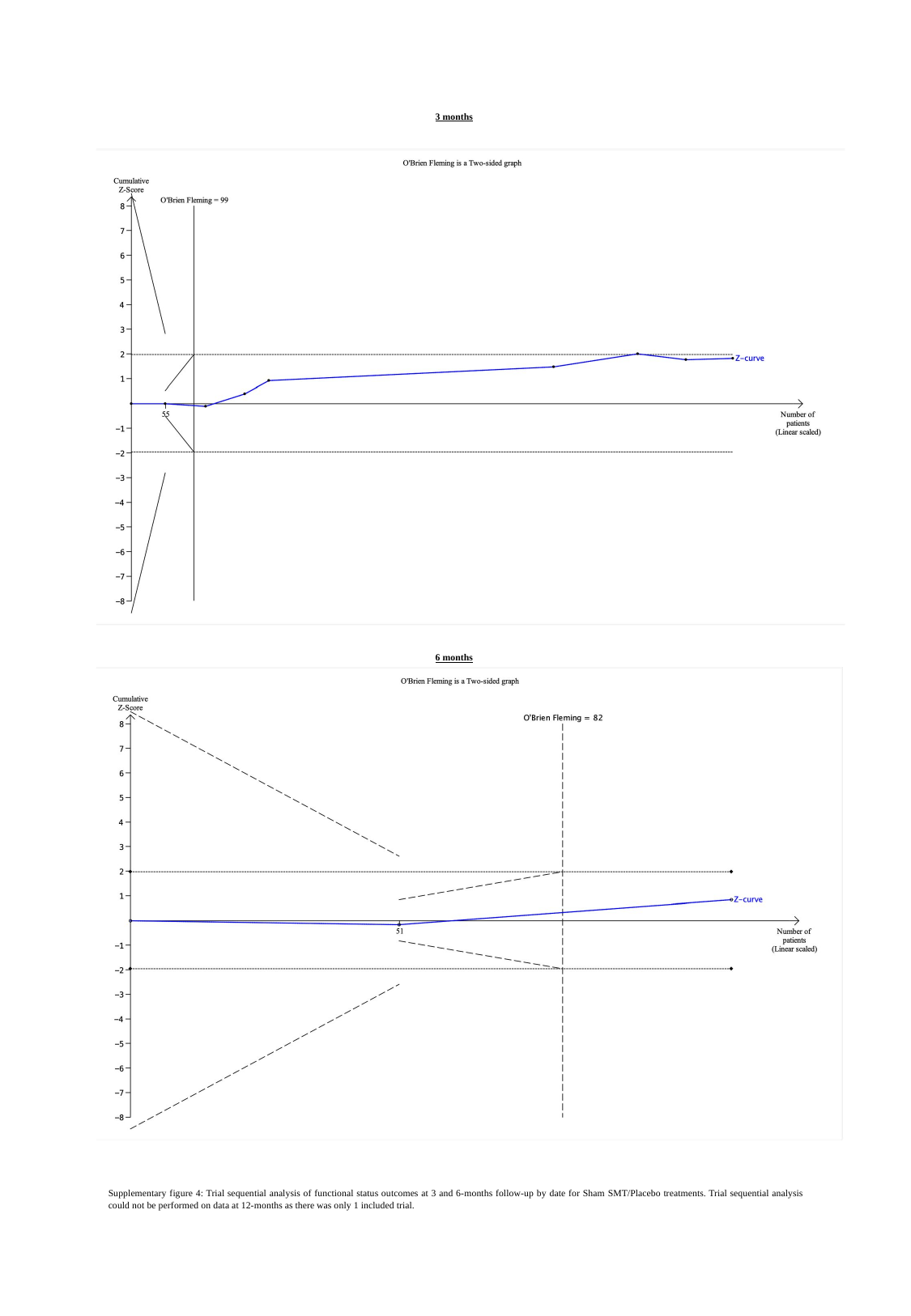

3 months
6 months
Supplementary figure 4: Trial sequential analysis of functional status outcomes at 3 and 6-months follow-up by date for Sham SMT/Placebo treatments. Trial sequential analysis could not be performed on data at 12-months as there was only 1 included trial.
